# Lord Derby's Grand Effort: The Medical Profession and Recruiting

**Published:** 1915-12-18

**Authors:** 


### LORD DERBY'S GRAND EFFORT.

### The Medical Profession and Recruiting.

The record of the effect of the appeal made to the young manhood of the country under Lord Derby's recruiting scheme, and especially the active response in the days just preceding the date fixed as a time limit, are experiences of vital interest to every citizen. They announce in a highly significant fashion the practically unanimous determination of the country to suffer any and every sacrifice in order to attain the triumph of the cause to which the nation has pledged its faith. Many of those who have now taken their position as soldiers of the King must be pushing to one side interests which are both dear to their hearts and important to their ambitions; and they are voluntarily undertaking risks which involve perils both to themselves and to others whose welfare is one of their most vital concerns. The men who have taken this step are worthy of our respect, gratitude, and admiration, and less must not be said of the households who share alike in the sacrifice and in the peril. For these latter the anxiety which is an inevitable consequence of the national situation now takes on an acutely personal quality, and the strain of parting and the danger of the adventure make multiplied homes in the country centres of individual foreboding and emotional strain. The pathos of the situation is hardly less conspicuous than its set resolve. Yet there is no other way if the quarrel we know to be just is to come to a good ending. The arbitrament of battle is none of our seeking, but as we may not escape it with honour we are bold to defend the right. Such is the plain lesson which the recruiting stations announce to all whom it may concern.

There is probably no group of men who so fully understand what this enormous increase of our armies means to the general body of the people as do those engaged in the practice of medicine. The marching troops and the armed men form one aspect of the picture, and it is these which are seen in newspaper statistics and are noted by the casual observer. On another aspect the door is shut, and only those whose mission carries them into the life and secrets of the home know by personal experience of the cares and anxieties which are there created and of the stress and strain which have patiently to be endured. It is the privilege of the doctor to be the family friend, and in not a few cases he, indeed, is the only friend. The claims upon him just now are numerous, and recent events will multiply them. Never is his ministry purely material, and in the present stress many will look to him for the word of comfort which makes for hopefulness and the courageous heart. In this respect there falls on the medical profession a special duty, for with none other rests a greater opportunity to encourage the steadfastness which is to be the great moral asset of the nation in the days of trial which it cannot be doubted are immediately before us. Not only those who bear arms must prove themselves good soldiers, but those also who with calm patience are called to stand and wait. To be able to encourage many to whom this difficult demand makes its appeal is no small thing, and, while the work carries neither glory nor wide recognition, it is none the less a real and substantial contribution to the triumph of the right. To labour this particular aspect of the great issue is the less necessary, for the temper of the nation in this hour of sacrifice leans not to personal distinction or the gain of applause, but to the silent and effective discharge of duty. This latter may be the sole reward, but it is enough and more than enough. In an appeal which is universal there is in some shape or other a place for each, and with this and with the opportunity which it brings is to be found a full ambition for all who seek honest service and desire no lesser aim.

If the recruiting returns mean increased opportunity in what may be called the ordinary ministry of medicine they speak with a special emphasis to the younger members of the profession. All these new soldiers shortly to be organised into new armies are leaving the work which has been the purpose of their lives and the homes which are dear to them. They have heard the appeal and have responded to it. To think of a failure to provide them due protection and guidance in the risks which must beset them is inconceivable. But a large part of this protection and guidance can be supplied only by members of the medical profession, and in the very nature of things the task falls mainly on the younger men. Just as plainly as is the appeal to the civilian, so is the appeal to the doctor. Indeed, to the latter the appeal is the stronger, for without his co-operation the readiness and sacrifices of the others will all be in vain. Armies cannot take the field inadequately supplied with medical officers, and hence the special obligation which falls at this juncture on the medical profession. To call upon men whose talent and industry have begun to bear practical fruit to sacrifice their positions and to postpone or even imperil their ambitions is indeed a hard thing, and no smooth words will minimise the seriousness of the position. But there is no alternative, or at least the only alternative is a paralysis of the nation's military endeavour. The issue is written in no uncertain terms, and the judgment of it may not be delayed. By the informed voices of all the authorities, military and medical alike, is announced plainly the need for more doctors. This need must be supplied, and it must be supplied both promptly and fully. The condition implies without a doubt much personal sacrifice, but in this respect it agrees with what is imposed in greater or less measure upon all. The time has come when this sacrifice must be made on behalf of the cause for which the nation has plainly shown itself to be steady and resolved. Who deems himself exempt must find a justification. The doctors, even as the civilians, must have their tribunal of appeal, and it is with this must lie the decision of each man's duty.

